# Electrolyte Disorders Induced by Antineoplastic Drugs

**DOI:** 10.3389/fonc.2020.00779

**Published:** 2020-05-19

**Authors:** Ignazio Verzicco, Giuseppe Regolisti, Federico Quaini, Pietro Bocchi, Irene Brusasco, Massimiliano Ferrari, Giovanni Passeri, Valentina Cannone, Pietro Coghi, Enrico Fiaccadori, Alessandro Vignali, Riccardo Volpi, Aderville Cabassi

**Affiliations:** ^1^Unità di Ricerca Cardiorenale, Clinica e Terapia Medica, Dipartimento di Medicina e Chirurgia (DIMEC), University of Parma, Parma, Italy; ^2^Unità di Ricerca sulla Insufficienza Renale Acuta e Cronica, Unità di Nefrologia, Dipartimento di Medicina e Chirurgia (DIMEC), University of Parma, Parma, Italy; ^3^Ematologia e Oncologia Medica, Dipartimento di Medicina e Chirurgia (DIMEC), University of Parma, Parma, Italy; ^4^Unità di Endocrinologia e Malattie Osteometaboliche, Clinica e Terapia Medica, Dipartimento di Medicina e Chirurgia (DIMEC), University of Parma, Parma, Italy

**Keywords:** electrolytes abnormalities, antineoplastic drug exposure, antidiuretic hormone (ADH), renal tubulopathies, tumor lysis syndrome

## Abstract

The use of antineoplastic drugs has a central role in treatment of patients affected by cancer but is often associated with numerous electrolyte derangements which, in many cases, could represent life-threatening conditions. In fact, while several anti-cancer agents can interfere with kidney function leading to acute kidney injury, proteinuria, and hypertension, in many cases alterations of electrolyte tubular handling and water balance occur. This review summarizes the mechanisms underlying the disturbances of sodium, potassium, magnesium, calcium, and phosphate metabolism during anti-cancer treatment. Platinum compounds are associated with sodium, potassium, and magnesium derangements while alkylating agents and Vinca alkaloids with hyponatremia due to syndrome of inappropriate antidiuretic hormone secretion (SIADH). Novel anti-neoplastic agents, such as targeted therapies (monoclonal antibodies, tyrosine kinase inhibitors, immunomodulators, mammalian target of rapamycin), can induce SIADH-related hyponatremia and, less frequently, urinary sodium loss. The blockade of epidermal growth factor receptor (EGFR) by anti-EGFR antibodies can result in clinically significant magnesium and potassium losses. Finally, the tumor lysis syndrome is associated with hyperphosphatemia, hypocalcemia and hyperkalemia, all of which represent serious complications of chemotherapy. Thus, clinicians should be aware of these side effects of antineoplastic drugs, in order to set out preventive measures and start appropriate treatments.

## Introduction

A series of electrolyte derangements can develop during treatment with anti-cancer drugs. While some of these alterations may be paraneoplastic (1), in many cases specific pharmacodynamic mechanisms can be identified impacting on fluid and electrolyte metabolism. Besides the possible occurrence of acute kidney injury, proteinuria, and hypertension (2), several antineoplastic agents can affect electrolytes tubular handling, as well as urinary water excretion by interfering with antidiuretic hormone (ADH). The aim of this review is to analyze in detail the mechanisms underlying the disorders of the metabolism of sodium, magnesium, potassium, calcium, and phosphate during anti-cancer drug treatment.

## Methods

An extensive review of English language literature was performed to identify all relevant articles describing the epidemiology, pathogenesis, preventive measures, and outcomes of electrolyte disorders induced by antineoplastic drugs. To this purpose, we searched PubMed, EMBASE™, CINHAL, Web of Science and Cochrane databases for relevant articles. Related search terms were used as follow: (“Hyponatremia”[Mesh]) OR “Diabetes Insipidus, Neurogenic”[Mesh]) OR “Diabetes Insipidus, Nephrogenic”[Mesh]) OR “Hypokalemia”[Mesh]) OR “Magnesium Deficiency”[Mesh]) OR “Hypercalcemia”[Mesh]) OR “Hypocalcemia”[Mesh]) OR “Hypophosphatemia”[Mesh]) AND “Antineoplastic Agents”[Mesh]).

Medical subject heading terms were used to enhance electronic searches. Additional studies of interest were identified by hand searches of references, and at least two reviewers independently reviewed each article for eligibility. Conference proceedings were excluded. The search was last updated on December 12, 2019. Nomenclature of drugs and their molecular targets conforms the recently published IUPHAR/BPS Guide to Pharmacology nomenclature classification (3).

## Physiopathology Of Electrolyte Disorders

Sodium (Na^+^) is the main cation of extracellular space, where it is actively extruded from the intracellular space by sodium-potassium ATPase. Total body Na content is pivotal in maintaining extracellular fluid and arterial volume, both related to tubular Na reabsorption and urinary excretion. Serum Na concentration (136-144 mmol/L) depends on the ratio of total body exchangeable Na and potassium to total body water (TBW). Serum Na changes are mainly related to TBW, which is regulated by ADH (4). Hyponatremia (Na<136 mmol/L) is classified as mild, moderate and severe degrees when serum Na level is between 130 and 135, 120 and 129 and lower than 120 mmol/l, respectively (1). In cancer patients, hyponatremia has an overall prevalence up to 47% with mild, moderate and severe degrees accounting for 36, 10, and 1% (5). Hyponatremia is as an independent risk factor for mortality and prolonged hospitalization (6, 7). According to extracellular fluid volume, hypovolemic, hypervolemic, and euvolemic hyponatremia can be distinguished (8). Hypovolemic hyponatremia results from a loss of TBW lower than total body Na and occurs in salt-wasting nephropathy, renal or extrarenal losses or adrenal insufficiency. Hypervolemic hyponatremia results from a rise of TBW greater than the excess of total body Na and occurs in either edematous disorders such as congestive heart failure, decompensated liver cirrhosis, nephrotic syndrome, or in end-stage renal failure. In edematous disorders, when effective arterial volume is reduced and ADH levels are high, the use of non-steroidal anti-inflammatory drugs (both non-selective and cyclooxygenase-2 selective drugs) can affect free water excretion and further increase ADH secretion and activity (9), determining or worsening hyponatremia (10). Euvolemic hyponatremia is the most frequent hypotonic disorder and presents with normal extracellular volume. The syndrome of inappropriate antidiuretic hormone secretion (SIADH) represents the classic euvolemic hyponatremia where ADH secretion, despite the hypo-osmolality state, is not suppressed and is potentiated by several drugs (antineoplastic treatments, antidepressant such as selective serotonin reuptake inhibitors, antipsychotics, anti-epileptics such as carbamazepine, oxcarbazepine, eslicarbazepine, sodium valproate, lamotrigine, levetiracetam, and gabapentin) (11) or alternatively is oversecreted as the expression of a paraneoplastic syndrome. Hyponatremia constitutes a potential complication of thiazide diuretic treatment through mechanisms affecting maximal urinary dilution ability, through stimulation of ADH release and ensuing euvolemic hyponatremia (12). Again, the simultaneous use of non-steroidal anti-inflammatory drugs can facilitate the development of hyponatremia (10).

Conversely, hypernatremia (serum Na > 144 mmol/L) occurs mostly as a result of an excessive loss of TBW relative to Na content, leading to a free water deficit (13). Hypernatremia (prevalence ~3%) is far less frequent than hyponatremia in hospitalized cancer patients (14).

Clinical manifestations of hypo- and hypernatremia are similar, and concern mainly the central nervous system: they are related to the severity and the rate of development of serum Na derangement (13). Both hypo- and hypernatremia can be associated with confusion, behavioral changes, headache, irritability, nausea and vomiting, lethargy, drowsiness/coma, seizures, and respiratory arrest.

Potassium (K^+^) is the main intracellular cation and is fundamental for resting membrane potential. Altered serum K concentration can modify the electrical activity of excitable cells (cardiac myocytes, skeletal muscle cells and vascular smooth myocytes), leading to serious adverse effects, such as life-threatening arrhythmias. Normal serum K levels range between 3.5 and 5.3 mmol/L. Chemotherapeutic agents may induce serum K derangements mainly through alterations of renal tubular transport. Hypokalemia (K < 3.5 mmol/L) prevalence is around 12% in cancer population patients (15); this figure increases between 43 and 64% in acute leukemia (16). Hyperkalemia (K > 5.3 mmol/L) is often related to tumor lysis syndrome (TLS) or to acute and/or chronic oliguric kidney disease.

Magnesium (Mg^++^) is the second most abundant divalent cation in the human body (1). It is mainly stored in bone, muscle, and soft tissues, and is important for neurotransmission, protein, and DNA synthesis, hormone-receptor interaction. Its normal serum concentration ranges between 1.6 and 2.6 mg/dL (0.65-1.07 mmol/L), and its homeostasis depends on intestinal absorption and renal excretion. In the kidney, transient receptor potential cation channel, subfamily M, member 6 (TRPM6), a Mg channel located in the apical cellular membrane of the thick ascending limb of Henle's loop and distal convoluted tubule, and exerts the rate-limiting step for Mg tubular transport. The activity of TRPM6 is regulated by the epidermal growth factor (EGF) and its receptor (EGFR) (17, 18). As TRPM6 and EGF/EGFR are mainly expressed in the distal convoluted tubule, this segment represents the main site of regulation of urinary Mg excretion. Mild hypomagnesemia can be pauci-symptomatic, whereas a severe disorder can represent a life-threatening condition. Symptoms involve cardiovascular system, with electrocardiographic alterations (prolonged QT interval), and the neuromuscular system with tremor, paresthesia, tetany, spasms, and seizures (1). Hypomagnesemia is also associated with reduced release and activity of parathyroid hormone (PTH) and reduced synthesis of active vitamin D and its receptors (19). Both hepatic 25-hydroxylation and renal 1α-hydroxylation of vitamin D leading to the active form of 1,25-dihydroxycholecalciferol are magnesium-dependent process (20) In addition, magnesium plays a central role in natural and adaptive immunity by interacting with vitamin D metabolites activity (21). In hospitalized cancer patients, hypomagnesemia (Mg < 1.5 mg/dL) has a prevalence around 17 % although antineoplastic drugs can increase this figure (e.g., up to 90% with cisplatin) (22, 23).

Calcium (Ca^++^) plays important roles in intracellular signaling, neurotransmission, membrane stability and bone metabolism. Its homeostasis is regulated by PTH via calcium-sensing receptors on parathyroid cells. PTH stimulates tubular Ca reabsorption, mobilizes Ca from bone and enhances the synthesis of 1,25-dihydroxycholecalciferol, which in turn increases Ca intestinal absorption, modulates PTH release and PTH-mediated bone demineralization. Serum total Ca concentration ranges between 8.5 and 10.5 mg/dL, with ionized Ca between 4.7 and 5.2 mg/dL (24). In hospitalized cancer patients, hypocalcemia (Ca < 8.5 mg/dL) has a prevalence around 13% (23). Generally, symptomatic hypocalcemia presents with irritability, tetany, psychosis, and prolonged QT interval (1). Ca levels should be checked in case of hypomagnesemia, because of a concomitant low PTH activity (22). Agents, such as platinum-compounds and anti-EGFR Monoclonal Antibodies (MoAbs), causing hypomagnesemia may also induce hypocalcemia (25). Hypercalcemia is far more common than hypocalcemia, ranging between 20 and 30% in patients with advanced cancer, in particular lung, breast and hematological malignancies (26). Three major mechanisms of hypercalcemia have been identified: (i) PTH-related peptide secretion by cancer cell, (ii) osteolytic lesion, (iii) 1,25-dihydroxycholecalciferol (calcitriol) secretion by the cancer cells.

Phosphate (${\text{PO}}_{4^{--}}$) is predominantly stored as an inorganic salt in bone as hydroxyapatite crystals. In plasma phosphate circulates both as an inorganic anion, or as an organic component of intracellular nucleic acids and cell membranes. Normal phosphate levels range between 2.5 and 4.5 mg/dL, and result from the balance between intestinal absorption, renal excretion, and release from the bone exchangeable fraction which is regulated by PTH, fibroblast growth factor 23 and calcitriol. Fibroblast growth factor 23 and PTH decrease phosphate serum levels by inhibition of tubular reabsorption. Conversely, calcitriol increases the intestinal absorption of phosphate and inhibits PTH secretion. Hypophosphatemia occurs as a result of phosphate redistribution between extra- and intracellular compartments, poor intestinal absorption, increased renal excretion, or prolonged hemodialysis/hemofiltration. Clinical manifestations include weakness, proximal myopathy, rhabdomyolysis, hemolytic anemia, and heart failure. Antineoplastic drugs may induce tubular damage and thus alter phosphate reabsorption, resulting in the development of hypophosphatemia. Proximal convoluted tubule dysfunction determines urinary wasting of phosphate, glucose, urate, and bicarbonate and leads to the acquired Fanconi Syndrome (FS). Phosphate levels lower than 2.5 mg/dL were reported in 49% of patients with advanced cancer whereas a lower fraction (23%) had phosphate levels less than 2.0 mg/dL (27). Hyperphosphatemia (phosphate > 4.5 mg/dL) is related to TLS, particularly in hematologic malignancies, and occurs more frequently as a consequence of chemotherapy than in spontaneous TLS (28).

## Antineoplastic Drugs

### Platinum-Derived Compounds

Platinum-derived drugs include cisplatin, carboplatin, oxaliplatin and nedaliplatin, and electrolyte disorders associated with these drugs are shown in Table 1. Nephrotoxicity represents the limiting factor of these drugs (2, 22). Compared with cisplatin and nedaliplatin, carboplatin and oxaliplatin appear to be less nephrotoxic and associated with less electrolyte derangements (34). Cisplatin nephrotoxicity results from cell damage in the S3 segment of the proximal tubule, distal convoluted tubules and collecting ducts (22). Electrolyte disorders are also related to cisplatin-induced DNA damage of thiazide-sensitive sodium-chloride co-transporter genes and to the apoptosis of distal tubule cells (34). Cisplatin treatment may cause hyponatremia through SIADH, related to both higher secretion of and sensitivity to ADH (32). Nausea and vomiting, which are common side effects of platinum-derived chemotherapy, are also powerful stimuli for ADH secretion. The incidence of hyponatremia can reach 59% (severe hyponatremia 12%) with cisplatin, whereas 20% is reported with carboplatin (29–31). Rarely, cisplatin-related hyponatremia may result from Renal Salt Wasting Syndrome (33). Hypernatremia can also develop with cisplatin due to acquired nephrogenic diabetes insipidus with ensuing hypotonic polyuria (34).

**Table 1:** Platinum derived drugs.

**Electrolyte disorder**	**Drug**	**Incidence (%) Type of study**	**Mechanism(s)**
Hyponatremia	Cisplatin Carboplatin	43-59 (B) (29, 30) 20 (C) (31)	SIADH; RSWS, DNA damage of the gene encoding the thiazide-sensitive chloride channel (29, 32–34)
Hypernatremia	Platinum-drugs	n.a.	Acquired NDI (32)
Hypokalemia	Cisplatin Carboplatin	27 (D,B) (31, 35)	Renal potassium wasting due to hypomagnesemia; Decreased intestinal absorption due to enterocyte cytoxicity (35, 36)
Hypomagnesemia	Cisplatin Carboplatin	56-90 (B, D) (22, 23, 37) 7-29 (D) (38–40)	Calcium-sensing receptor impairment; TRPM6/EGF pathway downregulation (18, 22, 41) Gitelman-like syndrome (42)
Hypocalcemia	Cisplatin Carboplatin	6-20 (B, D) (43) 16-31 (B, D) (43)	Impaired PTH release due to hypomagnesemia (24, 44, 45) Altered bone metabolism due to hypomagnesemia; Low vitamin D due to decreased 1-alpha-OHase activity (24, 43, 46)
Hypophosphatemia	Cisplatin alone (combined with C*yclophosphamide)*	10-77 (D) (47, 48)	Partial proximal tubular damage; Acquired FS (47, 49)

*Incidence and type of study column: the letter after the percentage indicates the type of evidence available: A isolated case; B case series; C pharmacovigilance notifications or registry; D observational study, clinical trial, metanalysis of clinical trials. n.a. not available. References in bracket square. FS = Fanconi Syndrome; NDI, Nephrogenic Diabetes Insipidus; PTH, Parathyroid hormone; RSWS = Renal Salt Wasting Syndrome; SIADH, Syndrome of inappropriate antidiuretic hormone secretion; TRPM6, Transient Receptor Potential Cation Channel, subfamily M, member 6/EGF = Epidermal Growth Factor*.

Platinum-derived agents can induce hypokalemia due to renal K wasting secondary to hypomagnesemia. The incidence of cisplatin-related hypokalemia is around 27% (35). Intracellular magnesium depletion reverts inactivation of voltage-dependent renal outer medulla K channels (ROMK), thus increasing kaliuresis. Increased distal Na delivery or elevated aldosterone levels are also required for exacerbating K wasting (36). Potassium supplementation may fail to correct hypokalemia until hypomagnesemia is corrected.

Hypomagnesemia is the most frequent electrolyte alteration caused by cisplatin and is related to its cumulative dose (50). Hypomagnesemia is associated with shorter survival (35, 50), and its incidence ranges between 56 and 90% of patients receiving cisplatin, being lower with carboplatin (22, 23, 37–39). It is mainly related to an impaired Mg reabsorption in the proximal tubule. However, cisplatin was shown to downregulate the TRPM6/EGF pathway resulting in Mg loss (41), and patients receiving platinum drugs can also develop persistent distal tubular dysfunction with a Gitelman-like syndrome characterized by hypocalciuria, hypomagnesemia and hypokalemic metabolic alkalosis (42).

Cisplatin long-term treatment can cause hypocalcemia in a dose-dependent manner (46); low-dose cisplatin combined with 5-Fluorouracil and interferon-alpha can also induce hypocalcemia (44). Frequently, hypocalcemia is associated with hypomagnesemia (45). Cisplatin can decrease 1-alfa-hydroxylation activity and result in low vitamin D3 levels. In cisplatin-treated patient, hypocalcemia incidence is around 6–20%, and is 16–31% in those treated with carboplatin (43).

Hypophosphatemia may often complicate treatment with platinum agents, with incidence reaching up to 77% (47, 48). Cisplatin causes proximal tubule cell apoptosis and necrosis, resulting in partial or complete acquired FS (49).

Considering the development of the individual ion disturbances, there are also differences related to the cumulative dose and to the cycles of chemotherapy schedule. In particular, among platinum-derived drugs, cisplatin-induced hyponatremia occurred in 50% of the patients after a median critical dose of 195 mg at cycle 2, while higher cumulative doses had to be administered to observe hypokalemia (560 mg at cycle 7). Median critical doses for development of hypomagnesemia and hypocalcemia in 50% of the patients were 160 mg and 240 mg at cycle 2 and 3, respectively, supporting the concept of a lower dose required to induce hypomagnesemia versus others ion disorders (35).

### Alkylating Agents

Table 2 shows the electrolyte disorders observed in patients treated with Alkylating agents. These drugs can frequently cause hyponatremia by impairing free water excretion. Hyponatremia usually occurs 12–48 h after the administration of cyclophosphamide (CYC). CYC can induce severe symptomatic hyponatremia through different mechanisms: (i) a tumor lysis-related SIADH due to massive release of ADH or ADH-like peptides from damaged tumor cells or normal pituitary cells. (56), (ii) an ADH-like activity of CYC metabolites on renal collecting tubules (57), (iii) an upregulation of vasopressin V2 receptors and aquaporin-2 channels through the suppression of IL-1 and TNF-α, leading to increased ADH effects (58), (iv) a CYC-induced nephrogenic SIADH with activation of vasopressin V2 receptors in absence of ADH stimulation (59). Although most cases (up to 89%) are related to intravenous treatment with single-pulse high-dose of CYC (51, 69), hypotonic hyponatremia was also reported after low-dose treatment (14% incidence) (51). High doses of CYC (30–40 mg/kg of body weight), moderate doses (20–30 mg/kg) and low doses (<20 mg/kg) can produce hyponatremia with different frequencies (51–53, 69). Indeed, as a half-saline hydration protocol is performed before and after CYC administration in order to force diuresis and minimize the risk of hemorrhagic cystitis, the administration of large volumes of hypotonic fluid can facilitate hypotonic hyponatremia during CYC treatment. Thus, isotonic solutions should be preferred as treatment. Several cases of SIADH-related hyponatremia have also been reported with chlorambucil, melphalan, busulfan, or ifosfamide (54, 60).

**Table 2:** Alkylating agents.

**Electrolyte disorder**	**Drug**	**Incidence (%) Type of study**	**Mechanism(s)**
Hyponatremia	*Cyclophosphamide* *Ifosfamide, Chlorambucil Busulfan, Melphalan*	Low dose 14; High dose 89 (D) (51–53) 15 (<120 mmol/L) (B) (54, 55)	Central SIADH; preventive infusion of hypotonic solutions (56) Upregulation of vasopressin V2 receptors (57, 58); Nephrogenic SIADH (59) SIADH (60)
Hypokalemia	*Ifosfamide* *Bendamustine*	15 (D) (61) 5 (<2.4 mmol/L) (D] (62, 63)	Proximal tubular damage (tubular acidosis, acquired FS) due to metabolite (chloroacetaldehyde) (34, 64, 65) Renal distal tubulopathy (acquired Giltelman syndrome) (63)
Hypophosphatemia	*Ifosfamide*	1-16 (41, 66, 67)	Proximal tubular injury (acquired FS) (68)

*Incidence and type of study column: the letter after the percentage indicates the type of evidence available: A isolated case; B case series; C pharmacovigilance notifications or registry; D observational study, clinical trial, metanalysis of clinical trials. n.a. not available. References in bracket square. FS, Fanconi Syndrome; SIADH, Syndrome of inappropriate antidiuretic hormone secretion*.

Ifosfamide (especially when combined with cisplatin) may induce hypokalemia as a consequence of proximal or distal tubular acidosis, or acquired FS. Ifosfamide enters proximal tubular cells through organic cation transporter 2 and, after metabolization to chloracetaldehyde, induces glutathione depletion and lipid peroxidation (64, 65). Ifosfamide is also associated with proximal tubular injury, phosphaturia, and hypophosphatemia especially at cumulative doses greater than 60 g/m2: its incidence is between 1% (moderate dose, 1–1.5 g/m2 over 1–10 days) and 16 % (high dose, 3.33 g/m2, over 1–4), but can increase if patients are pretreated with cisplatin (47, 55, 68, 70).

Bendamustine, a chemotherapeutic agent designed to have both alkylating and antimetabolite properties, can induce hypokalemia (severe hypokalemia in around 5%) through a distal tubulopathy (acquired Gitelman syndrome) and a mild diuretic effect (62, 63). Special attention should be given to patients with pre-existing hypokalemia and if the cumulative dose of bendamustine exceeds 1,080 mg/m2 in the chemotherapy scheme (63).

### Target Therapies

Electrolyte disorders reported in target therapies-treated patients are described in Tables 3–5 part I, II, III. Hyponatremia is one of the most frequent derangement. According to a recent meta-analysis, the highest incidence (63.4%) of all-grade hyponatremia was observed in patients treated with Brivanib, a selective type 1 fibroblast growth factor receptor/vascular endothelial growth factor receptor-2 (VEGFR-2) antagonist combined with Cetuximab, a recombinant chimeric monoclonal antibody anti-EGFR (Her1). Hyponatremia was also frequent (31.7%) after monotherapy with Pazopanib, a multikinase inhibitor of VEGFR-2/platelet-derived growth factory receptor (PDGFR) (6). Lower incidence was seen with Afatinib, a protein kinase inhibitor inhibiting EGFR 2 (Her2) (1.7%) (6). Cediranib, a pan-VEGFR tyrosine-kinase inhibitor showed a 65% incidence of overall hyponatremia (72). The highest incidence of severe hyponatraemia (<120 mmol/l) was reported with cetuximab (34.8%), while the lowest incidence with gefitinib, an EGFR inhibitor (1.0%) (6).

**Table 3:** Target therapies (part I).

**Electrolyte disorder**	**Drug**	**Incidence (%) Type of study**	**Mechanism(s)**
Hyponatremia	*Cixutumumab* *Bevacizumab* *Ado-trastuzumab* *Ipilimumab, Nivolumab* *Nivolumab* *Icrucumab, Etaracizumab* *Volociximab* *Brivanib, Imatinib, Dasatinib,Cediranib Nilotinib,Sorafenib*, *Sunitinib, Gefinitib, Pazopanib, Afatinib, Bosutinib* *Temsirolimus, Everolimus* *Interferon-alpha*, *Levamisole, Pentostatine* *Interferon-alpha* *Elacytarabine*, *Interleukin-2*, *Eribulin mesylate* *Bortezomib*	25 (D) (71) n.a. (A) Brivanib and Cetuximab 63.4 (D); Pazopanib 31.7 (D); Gefitinib 1 (D) (6); Cediranib 65 (D); 35(<120 mmol/L) (72)	Blockade of IGF-1 receptor (71, 73) SIADH; Nephrotic Syndrome (69, 70) CSWS (74) Adrenal insufficiency due to autoimmune hypophysitis (75, 76) Interstitial nephritis, autoimmune adrenalitis (77, 78) SIADH (?) (79, 80) SIADH (34, 81–83) Aldosterone resistance (84, 85) SIADH (86–89) Hyperglicemia (90) Unclear (91–93) SIADH (?) (94, 95) TLS (96)

*Incidence and type of study column: the letter after the percentage indicates the type of evidence available: A isolated case; B case series; C pharmacovigilance notifications or registry; D observational study, clinical trial, metanalysis of clinical trials. n.a. not available. References in bracket square. CSWS Cerebral Sal wasting Syndrome; IGF-1, Insulin-like growth factor-1; SIADH, Syndrome of inappropriate antidiuretic hormone secretion; TLS, Tumor Lysis Syndrome*.

Cixutumumab, an anti-insulin-like growth factor receptor 1 antibody, induced hyponatremia in 25% of patients in a phase II safety evaluation study (71); the mechanism involves an increase of fractional sodium excretion (73). In patients treated with Icrucumab and Bevacizumab, recombinant human MoAbs against vascular endothelial growth factor receptor-1 and VEGF-A, hyponatremia emerged as a dose-limiting toxicity factor (126). Since Bevacizumab increases the risk of severe proteinuria (127), hypervolemic hyponatremia has also been proposed as a consequence of nephrotic syndrome. Trastuzumab Emtasine, inhibitor of EGFR 2 (Her2) signaling, at a dose of 3.6 mg/kg every 3 weeks, can induce true hyponatremia by a Cerebral Salt Wasting Syndrome as reported in a patient with breast cancer and brain metastasis who needed repeated hospitalizations because of severe hyponatraemia (74). Both Etaracizumab, a humanized monoclonal antibody against αvβ3 integrin receptor, and Volociximab, a chimeric monoclonal antibody directed against human α5β1 integrin, were also associated with severe hyponatremia (79, 80). Ipilimumab, a monoclonal antibody against cytotoxic T lymphocyte antigen-4, may induce hypophysitis leading to hypopituitarism, adrenocortical insufficiency and hyponatremia (75). Loss of the regulatory effects of cortisol on ADH release may induce SIADH. Hyponatremia was also observed after Nivolumab and Pembrolizumab administration, two programmed-death-1 pathway inhibitors. Nivolumab-related hyponatremia can also involve SIADH-independent mechanisms, causing a true Na depletion such as in autoimmune hypophysitis with an isolated ACTH deficiency and secondary adrenal insufficiency (76), or a tubulointerstitial nephritis (77), or an adrenalitis with primary adrenal insufficiency (78). The first case of nivolumab-induced adrenalitis resulting in primary adrenal failure with hyponatraemia was described in a 43-year-old man that started nivolumab (3 mg/kg) at two weekly intervals, whose serum sodium dropped to 127 mmol/L after four cycles (78).

Tyrosine Kinase Inhibitors (TKI) treatment has been associated with SIADH. Hyponatremia-related SIADH was reported in patients affected by Bcr-Abl acute lymphoblastic leukemia treated by Imatinib at a dose of 400 mg/day, and was observed after 11 months of therapy (81), in those affected by refractory chronic myelogenous leukemia treated with Dasatinib (100 mg/day), Nilotinib (400 mg twice/day) and Bosutinib (500 mg/day) (82), as well as in patients with renal carcinoma or with metastatic head and neck cancer treated with Sorafenib (400 mg twice/day for a median duration of therapy of 3.4 months) (83).

Mammalian targets of rapamycin (mTOR) inhibitors. Different grades of hyponatremia have been reported with Temsirolimus and Everolimus, either in monotherapy or in combination, with aldosterone resistance being suggested as the main pathogenetic mechanism (84, 85).

Immunomodulators. Serum Na derangements have been reported with Interferon therapy in the past 2–3 decades (86). Hyponatremia was described after 7 days of Interferon treatment in a patient in therapy with carbamazepine, supporting an additive effect of both drugs on SIADH development (87). A case of translocational hyponatremia due to hyperglycemia was reported in a patient with Interferon-induced diabetes mellitus (90). Levamisole and Pentostatin also appear to be associated with hyponatremia, presumably via SIADH (88, 89). Different degrees of hyponatremia were also observed in patients treated with Eribulin mesilate, Elacytarabine and recombinant Interleukin-2 (91–93), although the mechanisms are not clarified.

Proteasome inhibitor. Hyponatremia secondary to SIADH is not frequent in multiple myeloma even though bortezomib treatment, alone or associated with dexamethasone, was reported to increase ADH secretion and effect (94). In a case report, the finding of the ADH-positivity of monoclonal plasma cells at immunohistochemical analysis, suggested TLS as the pathogenetic mechanism (96). In another case report, a patient treated for multiple myeloma developed SIADH-related hyponatremia 3 cycles after starting 1.3 mg/m2 bortezomib (95).

Treatment with MoAbs and Target Therapies can affect potassium metabolism (Table 4, part II). Cetuximab and other anti-EGFR agents decrease potassium levels through an impairment in magnesium balance (99). Combined grade 3 and 4 hypokalemia (serum K between 2.5–3.0 mmol/L, and <2.5 mmol/L, respectively) had an incidence of 6.2%, and all-grade hypokalemia of 8.0%, in cetuximab-treated patients (97). The anti-HER3 and anti-HER2 antibodies lumretuzumab and pertuzumab combined with paclitaxel induced grade 3 hypokalemia in 40% of patients with breast cancer (98). Bevacizumab can induce hypokalemia due to proximal tubular damage (100). Hypokalemia is also reported with Tremelimumab, Blinatumomab and Eribulin mesylate through an unclear mechanism, possibly via drug-induced diarrhea (102–104). Novel TKI as Volasertib (105) and mTOR inhibitors, particularly Everolimus (101), were associated with hypokalemia.

**Table 4:** Target therapies (part II).

**Electrolyte disorder**	**Drug**	**Incidence (%) Type of study**	**Mechanism(s)**
Hypokalemia Hyperkalemia	Cetuximab, Panitumumab Lumretuzumab, Pertuzumab (combined with paclitaxel) Bevacizumab Temsirolimus, Everolimus Tremelimumab, Blinatumomab, Volasertib, Eribulin Mesilate DRUG-INDUCING-TLS (MoAbs, TKI, PI, CAR-T) IMMUNOMODULATORS (Thalidomide, Lenaldomide)	6 (<3 mmol/L) (D) (97) 8 (all grade) (D) (97) 57 (all grade) (D); 40 (<3.0 mmol/L) (D) (98) n.a. n.a.	Renal potassium wasting due to hypomagnesemia (97, 99) Drug-induced secretory diarrhea (98) Proximal tubular damage (100) Acquired FS (101) Unclear; Possible drug-induced diarrhea (102–105) TLS (34, 101)
Hypomagnesemia	Cetuximab, Panitumumab Zalutumumab, Nimotuzumab Cetuximab (combined with irinotecan) Lumretuzumab,Pertuzumab (combined with paclitaxel)	2-6 (<0.9 mg/dl) (D) (99, 106) 5.9 (<0.9 mg/dl) [D] (107) 34; 3% (<0.9 mg/dl) (D) (98, 99, 106)	Renal magnesium wasting due to TRPM6/EGF/EGFR blockade (99, 108) Drug-induced secretory diarrhea (98)

*Incidence and type of study column: the letter after the percentage indicates the type of evidence available: A isolated case; B case series; C pharmacovigilance notifications or registry; D observational study, clinical trial, metanalysis of clinical trials. n.a. not available. References in bracket square. CAR-T, Chimeric Antigen Receptor-T; FS= Fanconi Syndrome; MoAbs, Monoclonal Antibodies; PI, Proteasome Inhibitors; TKI, Tyrosine Kinase Inhibitors; TLS, Tumor Lysis Syndrome; TRPM6, Transient Receptor Potential Cation Channel, subfamily M, member 6/EGF, Epidermal Growth Factor*.

Conversely, hyperkalemia has been observed in cancer patients as a consequence of TLS, sepsis, and adrenal insufficiency due to metastatic disease (Table 4, part II). Other mechanisms include the suppression of insulin release, reducing potassium intracellular uptake, in Octreotide-treated patients, as well as transcellular potassium shift or TLS in Thalidomide-treated patients (at doses ranging between 100 and 400 mg/die) (36). TKI Axitinib induces hyperkalemia through TLS development and distal tubular dysfunction such as in hyperkalemic type 4 renal tubular acidosis (128).

Electrolyte imbalances increased considerably after the introduction of anti-EGFR MoAbs into therapy (129) (Table 4, part II). A recent meta-analysis of 25 randomized controlled trials reported an incidence of hypomagnesemia of 34%, whereas those of hypokalemia and hypocalcemia were 14 and 17%, respectively. Cetuximab increased 6 times the risk of grade 3/4 hypomagnesemia (Mg serum between 0.7-0.9 or <0.7mg/dl, respectively) and grade 3/4 hypokalemia (17, 99, 130). The longer half-life and higher affinity of panitumumab for EGFR, as well as the overexpression of EGFR, are responsible for a high incidence of grade 3/4 hypomagnesemia and hypokalemia in colorectal cancer patients (99). Patients treated with cetuximab 400 mg/m2 at first dose and 250 mg/m2 weekly (or 500 mg/m2 every 2 weeks) or panitumumab 6 mg/kg (or 9 mg/kg according to the tumor types) developed hypomagnesemia and hypokalemia (99). Compared to cetuximab and panitumumab, zalutumumab is associated with less hypomagnesemia and hypokalemia (108). A recent phase 2 trial showed 5.9% incidence of hypomagnesemia when Cetuximab and Irinotecan were co-administrered (107) (Table 4, part II). Hypomagnesemia, by upregulating ROMK activity with ensuing potassium loss, is involved also in anti-EGFR MoAbs-induced hypokalemia (36). Magnesium supplementation should be considered in patients undergoing treatment with Anti-EGFR MoAbs. Some authors suggest empirically prophylactic administration of Mg at the beginning of treatment (1) and oral preparations are preferred in mild hypomagnesemia, while intravenous administration for severe depletion.

Cetuximab and Panitumumab can also induce hypocalcemia related to the underlying hypomagnesemia (99). TKI Imatinib can cause hypocalcemia and muscle cramps in up to 40% of patients. In a young woman with abdominal gastrointestinal stromal tumor, Imatinib (400 mg/day) led to hypocalcemia after 5 months of treatment (109) (Table 5, part III). A direct effect on c-Kit tyrosine kinase receptors of renal tubular cells with ensuing hypocalcemia represents the possible mechanism (110). Osteoclast inhibition and osteoblast activation with bone sequestration of Ca and phosphate and ensuing hypocalcemia may also be involved (111). Sorafenib (112), as well as Axitinib (114) and combined therapy with Erlotinib and Sunitinib (115), can cause hypocalcemia. A case of severe symptomatic hypocalcemia related to Nilotinib was also reported, with an immune-mediated destruction of the parathyroid glands or a drug interference with calcium sensing receptors (CaSRs) and ensuing insufficient PTH secretion being the suggested pathogenic mechanisms (113).

**Table 5:** Target therapies (part III).

**Electrolyte disorder**	**Drug**	**Incidence (%) Type of study**	**Mechanism(s)**
Hypocalcemia	*Cetuximab*, *Panitumumab* *Lumretuzumab, Pertuzumab* *(combined with paclitaxel)* *Imatinib* *Sorafenib* *Nilotinib* *Erlotinib, Axitinib, Sunitinib*	17 (D) (99) 14 (D) (98) 40 (A,B) (109)	Hypomagnesemia-related hypoparathyroidism (99) Direct effect on tyrosine kinase c-Kit of tubular cells (109); low-voltage-activated T channels blockade (110, 111) Endoplasmic reticulum stress with calcium mobilization (112) Immune-mediated parathyroid glands destruction; interference with CaSRs (113) Unclear (114, 115)
Hypophosphatemia	*TKI* *Sorafenib Combined with Capecitabine* *Vemurafenib, Dabrafenib* Proteasome Inhibitors *(Bortezomib, Oprozomib* *Carfilzomib)* Lenalidomide mTOR inhibitors *(temsirolimus)* MoAbs *(Nivolumab, Bevacizumab*, *Etaricizumab)* *Lumretuzumab, Pertuzumab (combined with paclitaxel)* *Mirvetuximab Soravtansine*	23 (<2.0 mg/dl) (D) (116) 40 (A, B) (109) 2.3 (<2.0 mg/dl) (D) (117) 17(D) (98) 25 (<2.0 mg/dl) (D) (118)	Bone Turnover inhibited; proximal tubule damage by PDGFR blockade (119, 120) Vitamin D malabsorption due to drug-induced secretory diarrhea (121) Acquired FS (120, 122) Acquired FS (123) Acquired FS (?) (124) Phosphate wasting due to acute tubular necrosis (34) Acquired FS (?) (79, 100, 117) Vitamin D malabsorption due to drug-induced secretory diarrhea (98) Vitamin D malabsorption due to drug-induced secretory diarrhea (118)
Hyperphosphatemia	*MoABS (Brentuximab, Obinutuzumab,Otlertuzumab, Ibritumomab,Ofatumomab)* *TKI* *Proteasome Inhibitors* *Lenalidomide and CAR-T cell*	n.a.	Tumor Lysis Syndrome (28, 125)

*Incidence and type of study column: the letter after the percentage indicates the type of evidence available: A isolated case; B case series; C pharmacovigilance notifications or registry; D observational study, clinical trial, metanalysis of clinical trials. n.a. not available. References in bracket square. CAR-T, Chimeric Antigen Receptor-T; CaSR=calcium sensing receptor; c-Kit, type III receptor tyrosine kinase; FS= Fanconi Syndrome; MoAbs, Monoclonal Antibodies; mTOR= mammalian target of Rapamycin; PDGFR, Platelet Derived Growth Factor Receptor; PTH, Parathyroid hormone; TKI, Tyrosine Kinase Inhibitors*.

TKIs (Imatinib, Sunitinib, Ceritinib) affect also phosphate balance via inhibition of bone turnover (Table 5, part III) (119, 131). TKI may affect bone turnover by inhibiting PDGFR in proximal tubular cells and induce, as in the case of imatinib, an acquired FS (120). Hypophosphatemia can be worsened by concomitant conditions, as in the case of sorafenib-induced diarrhea with consequent vitamin D malabsorption (121). Novel target therapies may also induce different grades of hypophosphatemia. Vemurafenib and rarely, Dabrafenib, both competitive inhibitors of mutated BRAF kinase, may cause acute (1–2 weeks) or subacute (1–2 months) tubular toxicity. Acute toxicity is associated with acquired FS while subacute toxicity to an immuno-allergic interstitial nephritis (122). Diarrhea-induced vitamin D malabsorption can be also responsible for hypophosphatemia in 25% of patients treated with Mirvetuximab Soravtansine, a folate receptor α-targeting antibody-drug conjugate (118). Finally, acquired FS was also reported in patients treated with Perifosine, a protein kinase B inhibitor (132), Lenalidomide (after three weeks at a dose of 15 mg/day) (124), proteasome inhibitors (123), mTOR inhibitors (34), and MoAbs (Nivolumab, Etaracizumab, Bevacizumab, with nivolumab showing a 2.3% incidence of grade 3 [<2.0 mg/dl] hypopophatemia) (79, 100, 117) (Table 5, part III). Among proteasome inhibitors, hypophosphatemia development was related to subcutaneous administration of 1.3 mg/m2 bortezomib during a 21-day cycle for 16 cycles, combined with thalidomide, dexamethasone, and panobinostat (133).

Finally, a special emphasis must be given to various target therapy drugs (Table 4, part II and Table 5, part III) causing diarrhea, a mechanism leading to a combination of electrolyte derangements including hypokalemia, hypomagnesemia, hypocalcemia, hypophosphatemia, normal anion gap (hyperchloremic) metabolic acidosis due to bicarbonate loss, and severe hypovolemia (102–104, 118, 121). Target therapy, and in general chemotherapy, can cause nausea and vomiting. Excessive vomiting, especially over a prolonged period of time, leads to hypovolemia and hypochloremic metabolic alkalosis due to loss of chloride and hydrogen ions that can be associated with hypokalemia and hypomagnesemia (134).

## Vinca Alkaloids

Vinca Alkaloids, including vincristine, vinblastin, and vindesine, may induce hyponatremia via SIADH, that commonly occurs 1–3 weeks after drug administration (range 2–21 days). The incidence of moderate-severe hyponatremia is around 11% (135) with vincristine, but a higher incidence was reported with vinblastine after 7 days of treatment (136) or even earlier (after 36 hours) after a variable vinblastine dose between 0.2 and 6 mg/m2/day (137).

SIADH can result from a direct neurotoxic effect on the hypothalamus with altered control of ADH secretion (138). Interestingly, antifungal azole therapy, that inhibits Vinca Alkaloids metabolism, leads to severe neurotoxicity and SIADH-related hyponatremia (139). Hypocalcemia has also been reported in Vinka alkaloyds-treated patients but its incidence is not known; the involved mechanism is the impairment of microtubule polymerization (140). Table 6 reports the electrolyte disorders associated with Vinka alkaloid agents.

**Table 6:** Miscellaneous.

**Electrolyte disorder**	**Drug**	**Incidence (%)** **Type of study**	**Mechanism(s)**
Hyponatremia	*VINCA ALKALOIDS (Vincristine, Vinblastin)* *ANTIMETABOLITES* *Methotrexate* *TOPOISOMERASE I inhibitor* *Irinotecan*	11-90 (D) (135–137) 6.6 (120-130 mEq/L) (D)(141)	SIADH (direct hypothalamic toxicity; potentiated by antifungal azoles) (138, 139) SIADH, CNS-derived natriuretic peptide secretion (142, 143) SIADH (32, 141)
Hypokalemia	ANTIMETABOLITES Methotrexate Pemetrexed Azacytidine		Impairment of ion channels of skeletal muscle myocytes; renal tubular acidosis (144) Acute tubular necrosis; tubular acidosis or acquired FS (145, 146)
	ANTIANDROGENS (Abiraterone) Octreotide	16.6-18 (D) (all grade) 2.6-4.4 (<3.0 mEq/L) (66, 147, 148).	17α-hydroxylase inhibition and accumulation of mineralocorticoids (149) Decreased cellular potassium uptake due to insulin suppression (34)
Hypocalcemia	VINCA ALKALOIDS (Vinblastine) ESTROGENIC AGENTS Estramustine ANTIBIOTICS Mithramycine, Actinomycin D, Actinomycin-F		Altered intracellular calcium homeostasis due to cell microtubular damage (118) Inhibition of PTH action on bone turnover (67, 150) Blockade of osteoclast function; resistance to PTH on bone turnover (151)
	ANTIMETABOLITES 5-Fluorouracil (combined with leucovorin) TRPV6 INHIBITOR (Soricidin 13)	65 (D) (152) 13 (D) (153)	Low vitamin D3 due to reduced 1-alpha-and 25-hydroxylase activities (152) Altered Calcium absorption (153)
Hypophosphatemia	ESTROGENIC AGENTS (Estramustine) NITROSUREAS (Streptozocin,Semustin,Carmustine, Lomustine) ANTIMETABOLITES Azacytidine		High phosphaturia due to down-regulation of NaPi-IIa, NaPi-IIc cotransporter in proximal tubule (150) Phosphate wasting due to -interstitial nephritis and tubular atrophy; FS (154) Proximal Tubule Damage (145, 146)
	HALICONDRIN ANALOGUE (Eribuline Mesylate) ANTIBIOTICS (Anthracyclines: amrubicin, doxorubicin)	8.6 (D) (155, 156) <2.0 mg (A) (157)	Unclear (155, 156) Proximal Tubule Damage (157)

*Incidence and type of study column: the letter after the percentage indicates the type of evidence available: A isolated case; B case series; C pharmacovigilance notifications or registry; D observational study, clinical trial, metanalysis of clinical trials. n.a. not available. References in bracket square. CNS, Central Nervous System; FS= Fanconi Syndrome; NaPi, Sodium-Phosphate cotransporters; SIADH, Syndrome of inappropriate antidiuretic hormone secretion; TRPV6, inhibition of member six of Transient Receptor Potential Vanilloid family of calcium channel*.

## Antimetabolites

Both increased secretion of ADH (142) or a central nervous system-derived natriuretic peptide (143) are described as potential mechanisms of Methotrexate-induced hyponatremia (Table 6). Methotrexate (at a dose of 12 g/m2) can induce severe hypokalemia, as observed in a patient with transient hypokalemic tetraparesis occurring after intravenous high-dose administration (144). Azacytidine (75 mg/m2/day administered subcutaneously or intravenously, days 1–7 of each 28-day cycle for 6 cycles) may also induce hypokalemia and hypophosphatemia; potassium depletion can persist for weeks after stopping the drug, and necessitates prolonged parenteral supplementation (145, 146). The combination of 5-Fluorouracil (at a dose of 425–600 mg/m2/day for 5 consecutive days) with 5-formyl tetrahydrofolic acid (leucovorin, 20 mg/m2/day) can induce hypocalcemia due to inhibition of vitamin D 1- and 25-hydroxylation, leading to low levels of calcitriol (Table 6) (152).

## Miscellaneous

Antiandrogens. Abiraterone inhibits both testicular and extra-testicular androgen synthesis by inhibiting 17α- hydroxylase and 17–20 liase resulting in decreased testosterone levels. The inhibition of 17α-hydroxylase leads to the accumulation of upstream mineralocorticoids that increase epithelial Na channel and the voltage-dependent ROMK activities in the distal nephron, resulting in increased cortical collecting duct potassium secretion and ensuing hypokalemia (149). The incidence of all-grade hypokalemia related to abiraterone (at a standard dose of 1,000 mg/day) ranges between 16.6 and 18% and between 2.6 and 4.4% when grade 3/4 hypokalemia (less than 3.0 mmol/L) is considered, and occurs after 2–4 weeks from the beginning of therapy (Table 6) (66, 149). Symptoms associated with mineralocorticoid excess and hypokalemia are managed by co-administration of low-dose prednisone, potassium chloride supplementation and/or a mineralcorticoid antagonist.

Estrogenic agents. Oral and intravenous administration of Estramustine (two 2,000 mg/m2 doses administered weekly) is associated with hypocalcemia and vitamin D3 deficiency after 4–5 weeks (67) (Table 6). Hypocalcemia may be due to inhibition of PTH action and impaired calcium mobilization from bone (67). Several studies reported phosphate wasting during high-dose estrogen treatments, hypothesizing a down-regulation of sodium phosphate co-transporters (NaPi-IIa and NaPi-IIc) in the proximal convoluted tubule through the activation of estrogen receptor-alpha, independently from the Klotho/FGF- 23 and PTH pathways (150) (Table 6).

Antibiotics. Mithramycin associates with hypocalcemia by blocking osteoclast function and PTH action on gut and bone directly, or by causing vitamin D resistance. Actinomicyn-D and Actinomycin-F also induce hypocalcemia by interfering with bone mineralization (151) (Table 6).

Nitrosureas. Streptozocin has a greater nephrotoxicity profile than other molecules, such as carmustine and lomustine. These drugs may induce renal toxicity through interstitial nephritis and tubular atrophy, resulting in FS (154).

Halichondrin B Analogue. Eribuline mesylate, a nontaxane inhibitor of microtubule dynamics has been associated with hypophosphatemia (around 8.6% in a phase I trial, at doses of 2.0 mg/m2/week, and with the liposomal formulation of the drug at doses between 1.0 and 3.5 mg/m2/week) (155, 156), but also with hyponatremia, hypomagnesemia, and hypokalemia (3–10%) (Table 6); the exact mechanisms of ion derangements have not yet been clarified.

TRPV6 Inhibitor. SOR-C13 reduces calcium intestinal absorption by the inhibition of member six of Transient Receptor Potential Vanilloid family of calcium channel (153).

## Tumor Lysis Syndrome

Tumor Lysis Syndrome often occurs as a consequence of cytotoxic therapies, mostly in patients affected by hematological malignancies, leading to rapid release of cell constituents. It can be complicated by life-threatening conditions such as cardiac arrhythmias, acute kidney injury and neurologic impairment (158). The electrolyte derangements observed in TLS include hyperphosphatemia, hyperkalemia, and hypocalcemia, with the latter being the result of calcium precipitation in soft tissues secondary to acute hyperphosphatemia. The highest incidence of TLS is observed after Dinaciclib and Alvocidib treatment (between 15 and 53 % in acute leukemia trials) whereas the incidence is 8–10% for Venetoclax, CAR-T cell, Obinutuzumab, and <5% with Brentuximab, Carfilzomib, Lenalidomide, Dasatinib and Oprozomib. Idelalisib, and Ofatumumab have no reported cases of TLS (125).

## First-Line Cancer Therapies And Ion Derangements

First-line therapy represents the regimen(s) that is(are) generally accepted for the initial treatment of a specific type and stage of cancer, and is intended to cure the tumor when possible. The strategy of treatment should consider several factors, such as histology and molecular pathology of cancer, and age and comorbidities of the patient in order to set adequate therapeutic decisions (159).

In 2018, according to World Cancer Research Fund International, breast, colorectal, lung, and cervical cancers are the most common cancers in the female population whereas lung, prostate, and colorectal cancers are the most common malignancies in the male population (160). Chemotherapy with platinum-based drugs (preferably cisplatin) is recommended in the majority of lung cancers, as a first-line treatment for locally advanced non-small cell lung cancer (NSCLC), small-cell lung cancer (SCLC), unresectable malignant pleural mesothelioma and in association with immunotherapy (checkpoint inhibitors such as Pembrolizumab, an inhibitor of programmed cell death protein 1 pathway) in metastatic NSCLC and in those positive for programmed cell death protein 1. In patients with NSCLC and with a sensitizing EGFR mutation, first-line therapy considers TKI (erlotinib, afatinib). The most frequent electrolyte disorders in patients treated with platinum-derived drugs are mainly hypomagnesemia and hyponatremia, with the latter being related to cisplatin, pembrolizumab or TKI combined effect. Less frequently patients can also show hypophosphatemia, hypocalcemia, and hypokalemia (161–165) (Tables 1, 3–5). Androgen deprivation therapy (Goserelin, a luteinizing hormone releasing hormone agonist) and Docetaxel, a semisynthetic taxane, are used as first-line treatments for advanced metastatic prostate cancer; in castration-resistant prostate cancer patients the treatment includes abiraterone, which can lead to resistant hypokalemia (Table 6) (166). Early colon cancer first-line treatment includes adjuvant therapy based on several regimens responsible for electrolyte derangements. Leucovorin in combination with 5-Fluorouracil can often induce hypocalcemia; the FOLFOX (Leucovorin, 5-Fluorouracil, Oxaliplatin) or CAPOX (Capecitabin, the prodrug of 5-FU + Oxaliplatin) regimens can induce hypomagnesemia, hyponatremia and hypocalcemia, but with lower frequency compared to Cisplatin (34, 167). First-line therapy in metastatic colorectal cancer consists in chemotherapy doublet by combining the above regimens with the anti-EGFR antibodies (in patients with wild-type RAS), or bevacizumab (in the case of mutated RAS), or in chemotherapy triplet (FOLFOXIRI: 5-Fluorouracil, leucovorin, irinotecan, and oxaliplatin) plus bevacizumab in case of BRAF mutation: hyponatremia, hypomagnesemia, hypocalcemia, hypokalemia, and hypophosphatemia are the ion disorders that these patients can experience (Tables 1, 6) (168). The most frequently chemotherapeutic regimens used in early estrogen receptor-negative breast cancer are represented by anthracyclines and/or taxanes, although in selected patients a CYC/Methotrexate/5-Fluorouracil combination can also be used. In Her2-positive patients, first-line therapy includes chemotherapy combined with anti-Her2 drugs as trastuzumab and pertuzumab. Triple-negative breast cancer are treated with chemotherapy alone. In early estrogen receptor-positive breast cancer, endocrine therapy should be added to the above-mentioned treatments (169). In advanced breast cancer anthracycline or taxane-based regimens, preferably as single agents, would usually be considered as first-line treatment for Her2-negative tumors. Capecitabine can also be a valuable option. In patients affected by Her2-positive advanced breast cancer first-line regimens include trastuzumab combined with vinorelbine or a taxane (170). Patients treated with a CYC/Methotrexate/5-Fluorouracil regimen can often experience hyponatremia because of the combined effects of Methotrexate and CYC, particularly when CYC is administered at higher doses. This disorder can be further amplified by combination with trastuzumab. The treatment with pertuzumab is complicated by secretory diarrhea leading to hypokalemia, hypomagnesemia and, due to vitamin D malabsorption, to hypocalcemia and hypophosphatemia. Hypocalcemia can also occur because of decreased calcitriol generation induced by 5-Fluorouracil or its prodrug Capecitabine; hypophosphatemia may develop, due to proximal tubule damage caused by anthracyclines (Tables 2–6) (152). Special attention should also be given to the presence of a concurrent hypokalemia and hypomagnesemia in patients following an anthracycline-based regimen; the cardiotoxic effects of these drugs can be amplified by the presence of these ion disturbances and be a harbinger of threatening arrhythmic events.

## Treatments Of Ion Derangements

Sodium: dysnatremias (both hypo- and hypernatremia), should be carefully evaluated for the cause (or causes) and treated with special attention to the timing of disturbance onset and the rate of correction. It is mandatory to know if the disturbance is acute (<48 h) or chronic (>48 h); in the case of acute hyponatremia with neurologic alterations, a rapid infusion of hypertonic saline is required to increase serum Na concentration by 1–2 mmol/L per hour. In chronic hyponatremia the correction rate should be 4–8 mmol/L per day, or even less (4–6-mmol/L per day) if there is a particularly high risk of osmotic demyelination syndrome. While the Adrogue-Madias equation can be used, when starting the infusion of a hypertonic solution, to predict serum Na concentration with therapy, several limitations have been described using this formula, and a close monitoring of the serum Na concentration is mandatory during treatment of patients with severe hyponatremia (171). In patients affected by SIADH and mild to moderate hyponatremia, fluid restriction is traditionally the first-line therapy; if fluid restriction is unsuccessful, pharmacological treatment with loop diuretics, urea, or vaptans should be considered.

In case of hypernatremia, sodium alterations should be corrected based on water deficit equation bearing in mind the importance of cause and the time of development of the electrolyte derangement and the rate of correction (13). Moreover, ongoing losses due to perspiration and urine output should be taken into account. The objective in patients with chronic hypernatremia is to lower sodium levels by a maximum of 10 mEq/L per day (less than 0.5 mEq/L/h is considered safe), whereas in those with acute hypernatremia the objective is to lower sodium levels by 1–2 mEq/L/h to restore normal levels in less than 24 h (172).

Other electrolyte disorders (K, Ca, Mg, P): the electrolytes depletions should be corrected with oral or intravenous supplementation. Treatment of K disorders in patients affected by malignancies is similar to that in the normal population. In case of hypokalemia and hypocalcemia secondary to hypomagnesemia, magnesium depletion should be primarily corrected (21). In case of acute hypercalcemia, in order to reduce calcium serum levels, an intravenous 0.9% saline infusion should be initially administered (usually 200 to 500 mL/h) since most patients are volume depleted. In the absence of renal or heart failure, loop diuretic therapy to increase urinary calcium excretion is not recommended; only if an impairment in the excretion of the fluid load is anticipated a loop diuretic should be considered. Bone calcium mobilization blockade with intravenous bisphophonates as zoledronic acid (a dose-adjustment in presence of renal failure is required) are frequently used in the treatment of acute hypercalcemia. Recently, treatment with denosumab, another antiresorptive drug with a long term effect, is increasingly being used. Patients affected by hypophosphatemia should receive medical treatment with vitamin D (calcitriol is preferred) and phosphate supplementation (1 to 3 g/day) (173).

## Conclusions

A vast array of traditional and novel antineoplastic drugs, currently available for cancer treatments, may induce serious and potentially life-threatening derangements in serum electrolyte concentrations, via mechanisms such as nephrotoxic tubular damage, diarrhea induction, and/or TLS. Oncologists and clinicians should be aware of these crucial side effects of antineoplastic therapies, in order to set out preventive measures and start promptly appropriate treatments when needed.

## Author Contributions

IV, GR, and AC: conceived and designed the review. IV, GR, FQ, PB, IB, MF, GP, VC, PC, EF, AV, RV, and AC: collected the data and contributed to the analysis of literature data. IV, GR, EF, and AC: performed the analysis of all data. IV, GR, FQ, and AC: discussion of the results. IV, GR, and AC: wrote the paper.

## Conflict of Interest

The authors declare that the research was conducted in the absence of any commercial or financial relationships that could be construed as a potential conflict of interest.
